# Complex preference relationships between native and non-native angiosperms and foraging insect visitors in a suburban greenspace under field and laboratory conditions

**DOI:** 10.1007/s00114-023-01846-9

**Published:** 2023-05-04

**Authors:** Scarlett R. Howard, Matthew R. E. Symonds

**Affiliations:** 1 Centre for Integrative Ecology, School of Life and Environmental Sciences, Deakin University, Burwood, VIC Australia; 2 School of Biological Sciences, Monash University, Clayton, VIC Australia

**Keywords:** Behaviour, Foraging, Invasive species, Halictid, Native bees, Pollinator

## Abstract

The introduction and spread of non-native flora threatens native pollinators and plants. Non-native angiosperms can compete with native plants for pollinators, space, and other resources which can leave native bees without adequate nutritional or nesting resources, particularly specialist species. In the current study, we conducted flower preference experiments through field observations and controlled binary choice tests in an artificial arena to determine the impact of field vs. laboratory methods on flower preferences of native bees for native or non-native flowers within their foraging range. We conducted counts of insect pollinators foraging on the flowers of three plant species in a suburban green belt including one native (*Arthropodium strictum*) and two non-native (*Arctotheca calendula* and *Taraxacum officinale*) plant species. We then collected native halictid bees foraging on each of the three plant species and conducted controlled binary tests to determine their preferences for the flowers of native or non-native plant species. In the field counts, halictid bees visited the native plant significantly more than the non-native species. However, in the behavioural assays when comparing *A. strictum* vs. *A. calendula*, *Lasioglossum (Chilalictus) lanarium* (Family: Halictidae), bees significantly preferred the non-native species, regardless of their foraging history. When comparing *A. strictum* vs. *T. officinale*, bees only showed a preference for the non-native flower when it had been collected foraging on the flowers of that plant species immediately prior to the experiment; otherwise, they showed no flower preference. Our results highlight the influence that non-native angiosperms have on native pollinators and we discuss the complexities of the results and the possible reasons for different flower preferences under laboratory and field conditions.

## Introduction 

Animal visitation to flowers for pollen transfer is important for successful pollination in many angiosperms. Approximately 300,000 animal species are involved in the pollen transfer (Kearns et al. 1998) of 87.5% of angiosperms (Ollerton et al. 2011), including bats, birds, bees, flies, beetles, moths, butterflies, wasps, and ants (Rader et al. 2016). Insects are one of the largest groups of animal pollinators, with bees being the most abundant, and arguably, one of the most important (Ballantyne et al. 2017). Research on the foraging behaviour of bees has predominantly focused on eusocial species, such as honeybees (see Giurfa et al. 1995; Greggers & Menzel 1993; Lowell et al. 2019; Moore et al. 1989) and bumblebees (see Essenberg et al. 2015; Lihoreau et al. 2016; Lunau 1991; Pasquaretta et al. 2019; Spaethe et al. 2001), despite eusocial bees representing only 1.5–2% of total bee species (Mortensen et al. 2015). The foraging behaviour and preferences of non-eusocial bees (e.g. solitary; subsocial; semisocial; quasisocial) are far less studied (De Araujo et al. 2020). Non-eusocial bees have been studied for their foraging behaviour under field conditions (Heard 1994; Stone et al. 1999; Welsford & Johnson 2012; White et al. 2001) and in foraging arenas (Dukas & Real 1991), but experiments and observations under more controlled laboratory conditions (Menzel et al. 1988; Welsford & Johnson 2012) are conducted far less, potentially due to the difficulty of capturing, keeping, and testing non-eusocial species (Howard 2021; Howard et al. 2021a).

Controlled behavioural studies with pollinators enable us to understand their learning, memory, vision, olfactory use, flower preferences, navigational abilities, and more. A major issue with these experiments is determining to what extent we can extrapolate controlled lab, greenhouse, and field-based behavioural studies to complex natural settings. Pollinator behaviour can be measured in different ways using a variety of frameworks, methods, and apparatuses. For example, insect behaviour can be tested and manipulated using different methods which range from fully immobilising the insect (e.g. Proboscis Extension Reflex [PER]: Devaud, et al. 2007; Giurfa and Sandoz 2012), to laboratory behavioural assays which constrain full movement (e.g. virtual reality environments: Rusch, et al. 2017; Buatois, et al. 2018;  Lafon, et al. 2021), to allowing free movement in natural environments (e.g. free-flying behavioural tests or observational studies).

Across the different experimental methods mentioned above, researchers generally have high manipulation of the environment and use controlled parameterised stimuli. Depending on what is being tested, different methods can provide valuable data on insect behaviour. However, the more constrained the insect is, the less relevant the results may be for natural situations. Field observations can provide a much more ecologically relevant perspective on plant-pollinator interactions, but are subject to less control of confounds and variability as well as sometimes yielding less quantifiable data. Therefore, there is value in combinations of experiments which can yield quantifiable results while being ecologically relevant enough to apply to real-world plant-pollinator interactions. Thus, in the current study, we further examine the disconnect in results between two methods by conducting flower-choice experiments through field observations and controlled behavioural experiments within small behavioural arenas. We then discuss the reasons for differences in results between the two methods.

Many studies and reviews have identified invasive plants as drivers of bee decline (for examples see: Batley & Hogendoorn 2009; Brown & Paxton 2009; Cardoso & Gonçalves 2018; Cardoso et al. 2020; Mathiasson & Rehan 2020; Stout & Morales 2009) and thus it is important to understand how native and non-native pollinators interact with these species and facilitate non-native plant reproduction. Non-native angiosperms can negatively impact native plants and increase competition between insect visitors trying to forage (Chittka & Schürkens 2001). The impact of invasive flora can be particularly harmful to specialist native bees (Drossart et al. 2017), which have evolved with native flora and generally forage on few closely related plant species or from a few species belonging to the same or related plant families (da Rocha-Filho et al. 2018). Furthermore, larvae of certain bee species cannot develop on the pollen from non-host plant species (Praz et al. 2008). Thus, examining the impact of invasive flora on the foraging behaviour of native bees is imperative for conservation efforts, urban planning, and park and garden design. Simply planting more flowering plants in an environment may not be adequate action to support, protect, and/or preserve native bees requiring resources such as pollen, nectar, sugar, and nesting materials (Requier & Leonhardt 2020).

Bees, though, are just one component of the pollinator fauna. The importance of insect pollinators other than bees can often be overlooked, despite contributing significantly to crop and native plant reproduction. Approximately 25–50% of global flower visits are made by non-bee insects (Rader et al. 2016). Importantly, 39% of visits to flowers of crops are performed by non-bee insects (Rader et al. 2016) with fruit sets increasing with visits from non-bee insects (Garibaldi et al. 2013; Rader et al. 2016). Thus, it is becoming increasingly important to study the plant-pollinator interactions of non-bee insects, alongside those of bees, to compare their behaviours, interactions, and their relative contributions to pollinating native and invasive plant species.

Biological invasions cause evolutionary change and cascading ecological consequences throughout environments. Australia is an apposite location for examining the relationships between pollinators and native vs. non-native plant species as it has a history of deliberate and accidental exotic flora and fauna introductions leading to widespread and significant ecological problems. Infamous examples include the cane toad (Shine 2010), the prickly pear (Freeman 1992), Paterson’s Curse (Zhu et al. 2017), and foxes (Saunders et al. 2010). Therefore, examining the impacts of non-native plants and how they may change or even damage native plant-pollinator systems is an important research area in Australia.

Australia is estimated to host over 2000 native bee species with at least 300–400 species yet to be discovered and described (Batley & Hogendoorn 2009; Leijs et al. 2018). There are five bee families found in Australia including Stenotritidae, Colletidae, Halictidae, Megachilidae, and Apidae. The family Stenotritidae and subfamily Euryglossinae are endemic to the region (Houston 2018; Michener 1965; 2007). Australia’s biogeographical isolation also means that the majority of its native bee species are found nowhere else in the world. In this study, we focus on observing the foraging behaviour and preferences of halictid bees (Hymenoptera: Halictidae) in comparison with other insects. We conduct further controlled behavioural experiments with *Lasioglossum (Chilalictus) lanarium* (Family: Halictidae), a native non-eusocial Australian halictid bee. This species is a ground nesting, generalist bee species, and is widespread across Australia (Atlas of Living Australia website n.d.). *L. lanarium* nests communally in aggregations of females and is known to forage on multiple flower lineages (Houston 2018).

In the current study, we first observed what plant species native halictid bees were foraging on and then conducted field surveys to record the abundance of insect visitors to the flowers of these plant species in a natural setting. We also captured native halictid bees (later identified as *L. lanarium*) from the flowers of these plants and tested them for their flower preferences in controlled laboratory conditions. If native halictid bees were observed visiting native plants more frequently in the field, we predicted that they would also select the flower of the native plant more frequently in binary choice experiments, if the laboratory experiments were an accurate test of flower choice in the field. Other factors could also influence bee choice such as differences in floral reward, flower colour, and competition. Field surveys and binary choice preference data are presented here to examine the foraging preferences and decision-making of a non-eusocial bee in a suburban green belt. We show that while these native bees preferentially visit flowers of a native plant in the field, these bees do not have a preference for the flowers of native plants in the behavioural experiments, and indeed may prefer visiting the non-native species, particularly if bees already have experience visiting flowers of that plant in the field. Our study also aimed to determine the ecological relevance of binary choice experiments between flowers of three plant species. If bee preferences differed between the two experiments, this suggests that changing the experimental methods impacts the results and thus these issues must be considered when designing plant-pollinator interaction studies. In particular, a difference in preference would suggest that binary choice experiments under controlled laboratory conditions did not reflect the real-world behaviour we observed in the field.

## Methods

### General procedure

Surveys and bee collection/release were conducted in a suburban greenbelt parkland in Wantirna South, Victoria, Australia (-37.868920, 145.201272) during the spring of 2020 between 11 am in the morning and 4 pm in the afternoon, when bees were observed to be most active. We first identified which flowers of different plant species halictid bees visited in the areas surrounding their nesting sites within nature strips. The collection and survey areas are surrounded by a conservation area to the west, public parks to the south, a vineyard to the south-east, a major road to the south, and surrounding livestock paddocks. All flowering plants in the area were observed to determine whether native halictid bees were visiting those flowers before experiments began. Halictid bees were observed visiting only three species of angiosperms in the area during spring September–November, which included one native plant species, *Arthropodium strictum* (Chocolate Lily), and two non-native plant species, *Arctotheca calendula* (Capeweed) and *Taraxacum officinale* (Common Dandelion). We conducted both field and laboratory observations of flower preferences including field surveys and behavioural choice observations in a laboratory environment. Surveys of insect visitation were conducted for flower patches of each of these three plant species. We then tested the preferences of individual halictid bees (*L. lanarium*) for flowers of native or non-native plant species after they were captured foraging on one of the three plant species (thus recording their immediate foraging history before behavioural testing). All insects visiting flowers were identified down to the lowest taxonomic level possible during observations.

### Field observations

Insect visitation to each of the flower species was measured for 15 min in 1.5 × 1.5 m quadrats. There were six quadrat observations for each of the three plant species resulting in a total of 18 quadrat observations before the plants died out due to heat exposure as the temperature increased closer to summer (specifically *A. strictum*). Each quadrat contained between 15 and 90 flowers depending on the plant species and day of the observations. Quadrats consisted of multiple plants of a single species in the areas near halictid nesting sites and were separated by at least 2 m. The plants were wild and grew in dense monospecific flower patches, thus making it possible for each quadrat to be restricted to a single species without removing any other plants or planting more of the selected species. *A. strictum* had a range of 35–75 flowers per quadrat with an average of 68 flowers, *A. calendula* had a range of 70–90 flowers per quadrat with an average of 87 flowers, and *T. officinale* had a range of 15–80 flower per quadrat with an average of 40 flowers. Each quadrat for a specific species was observed only once in a single day but quadrats for all three species were observed within a single day to account for weather and condition changes between days. The sequence of quadrat per species throughout each day was randomised. Data collection occurred over a 2-month period. Ambient temperatures ranged between 20 and 35 °C during the period of the study.

### Behavioural experiments

*Lasioglossum (Chilalictus) lanarium* (Family: Halictidae) bees were also collected from the field at the same time as quadrats observations were carried out. Bees were captured on one of the three plant species in small transparent plastic vials with air holes and transported inside of a dark opaque bag to a testing arena in a laboratory setting. Behavioural experiments were conducted on the day of capture during daylight hours and under natural lighting conditions. The plant species of the flower they were caught visiting was recorded (*A. strictum*: *n* = 10; *A. calendula*: *n* = 10; *T. officinale*: *n* = 16). A sample size of 10–15 bees per experiment is common in bee behavioural testing (see Giurfa et al. 1996; Chittka et al. 2003; Avarguès-Weber et al. 2020). Flowers used for the experiments were collected 1–3 h prior to tests by removing the flower with part of the stem connecting it to the rest of the plant. Bees were released following behavioural experiments in the same location as they were caught and were identified as *L. lanarium* before release and from past studies nests had been identified (Howard et al. 2021a; Howard 2021).

For experiments, bees were placed into a circular arena (20 cm in dimeter) with a neutral grey wall border (Howard et al. 2021a). Pilot experiments showed that bees were most likely to respond to flowers when placed within 10 cm of the flower. Thus, within the arena, bees were placed 5–10 cm away from flowers, equidistant to both, to motivate flower choice behaviour. A choice consisted of a touch of a flower in a binary choice paradigm, where bees were given the choice of the native flower vs. a non-native flower. A touch of a flower/stimulus is a common metric used to determine preferences for stimulus options in bee behavioural studies (Chittka et al. 2003; Giurfa et al. 2001; Perry and Barron 2013; Raine & Chittka 2008). After a choice, bees were removed from the flower with a toothpick and a new set of fresh flowers was placed into the arena. Flowers were changed between each trial to avoid scent marking cues by bees.

Bees were transported in and out of the arena and manipulated in the arena space with toothpicks. There were two flower comparisons consisting of the native species vs. the two non-native species (test 1: *A. strictum* vs. *A. calendula*; test 2: *A. strictum* vs. *T. officinale*). For each of the two tests, individual bees (*n* = 36) made 10 choices each as is common for recent tests we have conducted on *L. lanarium* (Howard 2021; Howard et al. 2021a). Flower species were randomised for side placement between each choice to account for side biases. All bees participated in both tests. Bees were given a break of 10–30 s between each choice. The testing sequence was randomised for each bee.

The flowers of plants used in the tests were those on which the halictid bees had been observed in the field. Flower size (diameter and surface area) was kept as similar as possible between flower types. Both characteristics could not be controlled for every pairing but were randomised for which species was larger, smaller, or when possible, flower size was kept the same.

### Statistical analysis

#### Field observations

To determine whether the total number of insects observed on the flowers of each plant species was significantly different, we analysed the data with a generalized linear mixed-effects model (GLMM) with a Poisson distribution with a log-link function including the plant species (categorical), number of flowers within the quadrat (continuous), an interaction between the plant species and flower number, and the intercept term as fixed factors and survey sequence (the sequence of all 18 surveys conducted to take into account time of day and survey sequence overall) as a random term to account for any sequence effects. The number of insects observed visiting flowers was used as the response variable. In order to determine what combination of predictors best explained number of insect visitors, we compared the AICc values from the different models (Anderson and Burnham 2004). The same analysis was employed for data including (i) only halictid bees and (ii) other insects which were not identified as halictid bees. We compared all possible models using the “dredge” function in the MuMIn package written for the R statistical language, run in R version 4.0.3 (Barton 2022).

#### Behavioural experiments

To determine whether there was a difference between the two choice tests and thus whether the tests needed to be analysed separately (choice test 1: *A. strictum* vs. *A. calendula*; choice test 2: *A. strictum* vs. *T. officinale*), we analysed the data by employing a generalized linear mixed-effects model (GLMM) with a binomial distribution including the test type (choice test 1 or 2—categorical) and the intercept term as fixed factors and individual bee ID (subject) as a random term to account for repeated measures. The model used a categorial response variable with two levels (a choice for a native species recorded a score of 1; a choice for a non-native species recorded a score of 0). The Wald statistic (*z*) tested if the mean proportion of native flower choices recorded, represented by the coefficient of the intercept term, was significantly different from chance expectation, i.e. H_0_: MPCC (Mean Percentage of Correct Choices) = 0.5.

If the two tests were found to be statistically different (see below), a GLMM was then used to determine whether bees preferred native or non-native flowers in the separate choice tests. We analysed the data from each choice test separately by employing a GLMM with a binomial distribution using only the intercept as a fixed factor and individual bee ID (subject) as a random term to account for repeated measures.

Both models were estimated using the routine “glmer” available as part of the “lme4” package written for the R statistical language, run in R version 4.0.3 (Bates et al. 2014, 2007; R Core Team 2020).

## Results

### Field observations

**Fig. 1:**
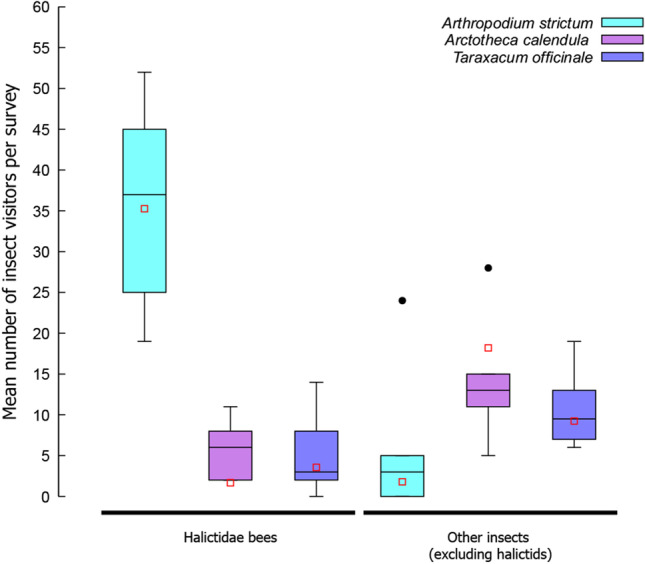
Boxplot of insect visitation counts for flowers over the six surveys for each plant, including *A. strictum* (cyan)*, A. calendula* (violet)*,* and *T. officinale* (blue). On the left are the number of halictid bees counted visiting each of the three flower patches while the right shows visits of all other insects to the three flower patches in surveys. Boxes show median (horizontal black line within box) and interquartile ranges with stems showing the main range of the data (excluding outliers). Closed black circles show outliers. Open red squares show the model predicted means for each insect group and plant species when number of flowers was set to 65 (average flower number across all quadrats) for comparison purposes

When comparing counts of halictid bees visiting patches of flowers of different species, the model that best fit the data was one that included the intercept and plant species of the flower (see Table 1). The number of halictid bees visiting flowers was significantly influenced by the plant species of the flower. Significantly more halictid bees were recorded visiting flowers of the native *Arthropodium strictum* than *Taraxacum officinale* (*z* = -9.981; *P* < 0.001) and *Arctotheca calendula* (*z* = -10.127; *P* < 0.001; Fig. 1).

**Table 1:** Model selection information for the different models best predicting halictid bee counts on flowers including the intercept, plant species, and flower quantity as variables

	Intercept	Plant species	Flower quantity	Interaction between plant species and flower quantity	df	logLik	AICc	Delta	Weight
2	3.560	+			4	-63.290	137.7	0.00	0.829
4	3.989	+	-0.0063170		5	-62.932	140.9	3.21	0.167
8	3.533	+	0.0004593	+	7	-61.484	148.2	10.51	0.004
3	2.384		0.0051530		3	-172.420	352.6	214.90	0.000
1	2.725				2	-174.321	353.4	215.78	0.000

When comparing counts of other insects (excluding halictid bees) visiting patches of flowers of different species, the model that best fit the data was one that included the intercept and plant species of the flower (see Table 2). There was a significantly greater number of insects (excluding halictid bees) visiting the flowers of both non-native species, *A. calendula* (z = 6.199; *P* < 0.001; Fig. 1) and *T. officinale* (z = 5.062; *P* < 0.001; Fig. 1), compared with flowers of the native species, *A. strictum*.

**Table 2:** Model selection information for the different models best predicting insect counts (other than halictid bees) on flowers including the intercept, plant species, and flower quantity as variables

	Intercept	Plant species	Flower quantity	Interaction between plant species and flower quantity	df	logLik	AICc	Delta	Weight
2	0.9079	+			4	-53.366	117.8	0.00	0.866
4	0.7307	+	0.002570		5	-53.275	121.6	3.74	0.133
8	0.5846	+	0.004646	+	7	-53.206	131.6	13.80	0.001
1	2.2010				2	-81.575	167.9	50.14	0.000
3	1.9400	0.003953			3	-80.887	169.5	51.68	0.000

*Arthropodium strictum* received the most visits from insects, largely driven by visitations from halictid bees which made up 93.47% of total visits. Other visiting insects to *A. strictum* included a single hoverfly, a single dragonfly, a single species of Coccinellidae, and multiple Lepidoptera species, among other insects. Halictid bee visits to *A. calendula* made up 29.17% of total visits and was the most common insect visitor, with other insect visitors including honeybees (*A. mellifera*), native wasps, non-native wasps, Lepidoptera, flies, hoverflies, and ants, among others. Visits by halictid bees to *T. officinale* made up 31.91% of visits by insects, which was the most common insect visitor, closely followed by hoverflies which made up 29.79% of visits. Other insect visitors included honeybees, a single native wasp, flies, and Lepidoptera, among others.

### Behavioural experiments

**Fig. 2:**
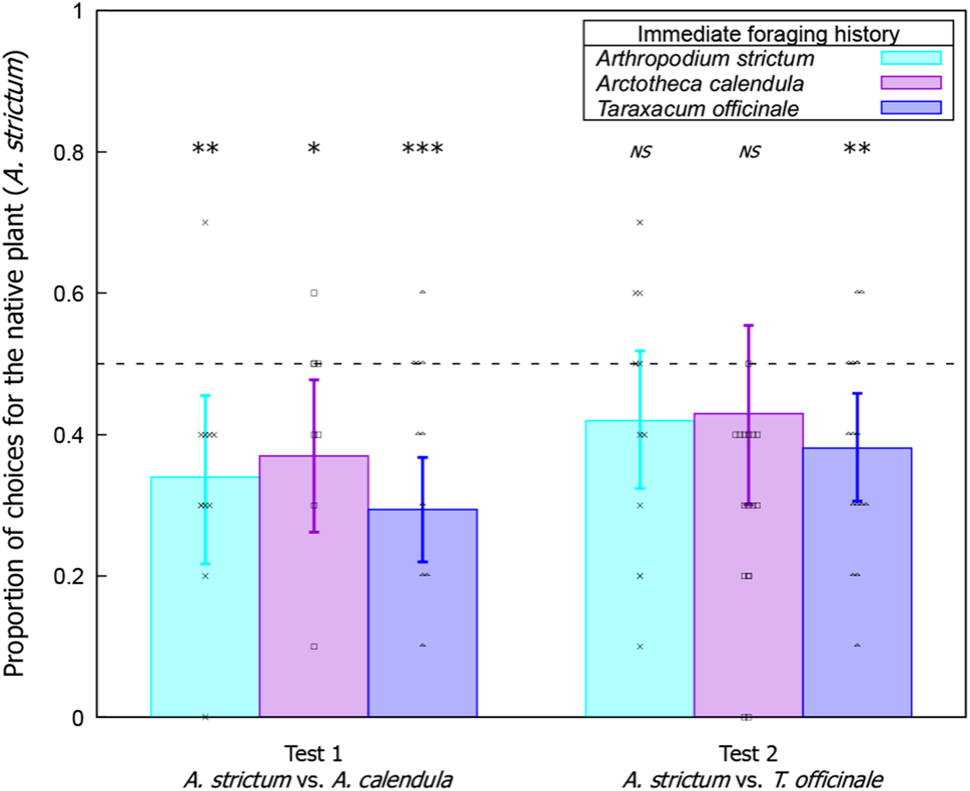
Proportion of choices for the native flower, *A. strictum*, during the preference testing for test 1 (*A. strictum* vs. *A. calendula*) and test 2 (*A. strictum* vs. *T. officinale*). In each cluster, the flower preferences of bees collected while foraging on *A. strictum* (cyan column; *n* = 10), *A. calendula* (violet column; *n* = 10), and *T. officinale* (blue column; *n* = 16) are shown. Dashed line at 0.5 indicates chance level performance (i.e. no preference for either plant species). Crosses, (*A. strictum*) open squares (*A. calendula*), and open triangles  (*T. officinale*) show individual choice data from each test. Significance from chance level performance is indicated by *P < 0.05, **P < 0.01, and ***P < 0.001. *NS* indicates a result that is not significant from chance level. Data shown are means ± 95% CI boundaries for all tests

The two choice tests yielded significantly different results (*z* = 2.162; *P* = 0.031) and were thus analysed separately. In choice test 1, *A. strictum* vs. *A. calendula*, bees originating from all flowers preferred the non-native *A. calendula* compared to flowers of the native plant species. See Table 3 and Fig. 2. In test 2, *A. strictum* vs. *T. officinale*, only bees originating from *T. officinale* showed a preference for that flower. Bees originating from *A. strictum* and *A. calendula* showed no significant preferences. See Table 3 and Fig. 2.

**Table 3:** Comparison of the generalised linear mixed models predicting flower choice in a binary choice assay with *Lasioglossum lararium*

Test type	Original flower on which the bee was collected	Mean preference for the native flower	Sample size	Confidence intervals	*z-value*	P-value
Test 1: *A. strictum* vs. *A. calendula*	*A. strictum*	0.340	10	0.217, 0.455	-2.777	< 0.006
	*A. calendula*	0.370	10	0.262, 0.477	-2.511	0.012
	*T. officinale*	0.294	16	0.220, 0.368	-5.054	< 0.001
Test 2: *A. strictum* vs. *T. officinale*	*A. strictum*	0.420	10	0.324, 0.518	-1.593	0.11
	*A. calendula*	0.430	10	0.301, 0.554	-1.228	0.220
	*T. officinale*	0.381	16	0.306, 0.458	-2.975	< 0.003

In summary, despite the results of the field surveys, controlled behavioural preference testing of *L. lanarium* showed that bees preferred flowers of the non-native plant species, *A. calendula*, compared to the native plant *A. strictum*, regardless of the immediate foraging history of the individual bee. Bees which were collected foraging on any of *A. strictum*, *A. calendula*, or *T. officinale* preferred flowers of the non-native *A. calendula* compared to the native *A. strictum* in binary preference tests. Interestingly, immediate foraging history impacted the results when we presented bees with the choice of *A. strictum* vs. *T. officinale*. Only bees which had been previously foraging on *T. officinale* preferred the flowers of this species, while bees that had been foraging on either *A. strictum* or *A. calendula* showed no overall preferences during the tests.

## Discussion

In the current study, we examined the flower preferences of insects, particularly native Australian halictid bees, for native and non-native plant species under field and laboratory conditions. During quadrat observations, we found that halictid bees regularly visited the flowers of three angiosperms: one native and two non-native species. Our surveys showed that halictid bees had the highest number of visits to flowers of the native plant, *A. strictum*. The survey results also showed that insect visitors other than halictid bees more often visited the flowers of the non-native plant species compared to the native species. Behavioural preference testing showed that bees had an overall preference for flowers of the non-native plant species, *A. calendula*, regardless of foraging history. We also found that when presented with *A. strictum* vs. *T. officinale*, bees only showed a preference for any of the flowers when their immediate foraging history was with *T. officinale* and in this case, they preferred this species.

A pertinent question raised in this study is why the apparent preference behaviour of halictid bees for flowers of native or non-native plant species changed between field and laboratory conditions. In the field, halictid bees were most abundant on the native flowers of *A. strictum*, but the results from the controlled preference testing showed a significant preference for flowers of the non-native *A. calendula*, regardless of whether they had been found foraging on native (*A. strictum*) or non-native (*A. calendula* or *T. officinale*) plants. A few potential hypotheses could explain the change in flower choice, besides the change from field to laboratory conditions. The first explanation is that *A. calendula* provides both nectar and pollen, while *A. strictum* provides only pollen through buzz pollination (Hogendoorn 2019), therefore perhaps at the time of experiments, after being both nectar and pollen deprived for 1–4 h, bees were inclined to prefer the non-native flower providing both nectar and pollen. Another potential explanation for the differing preferences in the field and under laboratory conditions is the impact of competition for floral resources from both native and non-native insects, particularly the introduced honeybee. As the flower of native *A. strictum* plants is buzz-pollinated, honeybees are unable to collect pollen from the flowers and thus we suggest this is why we observed no honeybees visiting this flower; however, honeybees were observed on flowers from both *A. calendula* and *T. officinale*. Honeybees are suggested to compete with native Australian bees for resources (Gross & Mackay 1998; Gross 2001; Hingston et al. 2004; Houston 2000; Prendergast et al. 2022; Sugden & Pyke 1991; Sugden et al. 1996; Threlfall et al. 2015). As *L. lanarium* preferred *A. calendula* to *A. strictum* in the preference test, halictid bees may also prefer *A. calendula* in the field, but due to competition with honeybees, and perhaps also with hoverflies, lepidopterans, and other insects, they were most abundant on the native flowers of *A. strictum* due to less competition with other insect species. However, this result is not consistent across tests, as when we presented bees with *A. strictum* vs. *T. officinale*, they only showed a preference for *T. officinale* when collected from this flower, even though it also provides both pollen and nectar. Thus, the floral rewards provided by flowers of each species could potentially explain some of the results, but not all preferences of bees, particularly in the test of *A. strictum* vs. *T. officinale*. It is likely that the preferences of bees are a combination of floral traits, availability, competition, floral reward type, and in the case of this study, experimental methods.

he observed preference of *L. lanarium* for flowers of the non-native plant, *A. calendula*, compared to flowers of the native plant, *A. strictum*, during binary choice experiments could also be due to differing flower characteristics. Bees are attracted to flowers through flower signals and traits including colour, scent, shape, pattern, and size. All of these flower characteristics differed between the flower types, with the exception of size which we attempted to control for in the current study. To a human observer, the colour of the flowers from the non-native plants was yellow while the native plant had a purple/violet coloured flower. Recent work has shown that *L. lanarium* has a preference for yellow-coloured stimuli in a controlled psychophysics test, although this could be an innate preference, or a preference acquired through experience foraging on certain flower colours (Howard et al. 2021a). An innate preference for yellow flowers could explain the choice for *A. calendula* over *A. strictum* in the binary choice experiment, even for bees originating on the purple flowers of *A. strictum*. The case of bees preferring *T. officinale* over *A. strictum*, when bees were collected from *T. officinale*, could be due to an acquired preference for yellow-coloured flowers. Similarly, *L. lanarium* has recently been shown to prefer certain flower morphologies, which are frequently pollinated by insects and generally appear star-shaped (Howard et al. 2021b), and this shape preference also occurs in honeybees (Lehrer et al. 1995; Howard et al. 2019, 2021). An innate or acquired preference for specific flower shapes may also have driven choice behaviour. Thus, we know from recent studies that *L. lanarium* have preferences for certain colours and shapes, which could have influenced their choices in the field or in the binary choice experiment. Other factors such as scent, flower density, or prior experience may have also had an impact on their flower choices, although we attempted to take these factors into account during the methods and analyses.

A major threat to bees as a result of urbanisation and the effect of human-influenced environments is the introduction or presence of non-native flora. In Australia, some studies have compared the impact of urbanisation and human-influenced environments on bee abundance and species richness. A study comparing bee communities in urban green spaces such as golf courses, public parks, and residential neighbourhoods showed that a lack of nesting habitat and dominance of introduced angiosperms in residential areas negatively impacted cavity and ground nesting specialist bee species such as Halictidae and Colletidae. However, these landscape characteristics, while negative for native bees, positively impacted the introduced honeybee, *A. mellifera* (Threlfall et al. 2015). A recent study examining the plant-bee visitor networks in urban remnant bushland and residential gardens showed that while the bushland had fewer total plant species, it had a higher proportion of native Australian flora. The results reflected that the introduced honeybee was associated more with the urban residential gardens, as well as native bee taxa including *Amegilla*, *Exoneura*, *Lasioglossum*, and *Homalictus* to a lesser extent. However, Euryglossinae, *Leioproctus*, and in particular *Megachile* were associated with native bushland remnants, which may be due in part to the presence of non-native flora in the urban areas (Prendergast & Ollerton 2021). Recent work in a rapidly expanding city, Melbourne, Australia, has shown that native bees tend to prefer areas of native remnant bushland while other insects such as Coleoptera and Lepidoptera were more frequently observed in urban residential areas (Shrestha et al. 2021). This suggests that the presence of native flora is important to support bees in urban regions. While this type of research in Australia is currently limited, the impact of human-influenced environments on Australian native bees shows that in general, native flowers are positively related to native bee abundance and richness (Threlfall et al. 2015), although this may change based on the specific bee taxa being examined (Prendergast & Ollerton 2021).

Studies have demonstrated that the flowers of invasive plant species can be very attractive to bees (for examples see: Buchholz & Kowarik 2019; Chittka & Schürkens 2001; Tepedino et al. 2008). While non-native plants can be incorporated into some bee diets (Stout & Morales 2009) and some pollinators may even come to rely on non-native flora (Vila et al. 2009), there are many negative consequences to both native bees and native flora of non-native plant introductions. Native plant fitness can suffer (Chittka & Schürkens 2001) when the flowers of non-native plants are more attractive to visit than native plants due to characteristics such as strong scents, high sugar production (Chittka & Schürkens 2001), and pollen nutrients (Drossart et al. 2017). The presence and relative attractiveness of non-native plant species can result in less pollinator visits to native plants (Aizen et al. 2008; Buchholz & Kowarik 2019; Chittka & Schürkens 2001; Morales & Aizen 2002; Tepedino et al. 2008), the disruption of pollination patterns of native plants (Montero-Castaño and Vila 2012), higher competition between pollinators and an increase in non-native pollinators (González-Varo et al. 2013; Morales & Aizen 2002), and a reduction in native plants (Chittka & Schürkens 2001; Stout & Morales 2009), among many other negative impacts on native pollinators and plants.

It should be noted that the results of our current study are based on a single environment and the controlled behavioural testing of a single native bee species which was observed visiting three plant species in a specific environment, a suburban greenbelt. The results of our study, while interesting, cannot be extrapolated to a wider range of environments, nor to other flora and fauna. More research is needed on a range of plant and pollinator species within different plant-pollinator networks across varied landscapes to examine the wider impact of non-native flora introduction and spread on bees.

Our study had a focus on the comparison between uncontrolled field observations and highly controlled behavioural experiments. Overall, more controlled experiments have many advantages, such as less noise, more control over variables and confounds, consistent data collection, accuracy of treatments, replicable results, and potentially larger sample sizes. However, they also lead to results which may be less generalisable and ecologically relevant. Where, field-based experiments can yield more ecologically applicable conclusions, which may more closely resemble what happens in real-world situations, it can be more difficult to control variables, collect data, replicate the experiment under the exact same conditions, and collect large data sets. Compromises such as conducting pollination and flower choice experiments in greenhouses can aid in this process but still suffer from issues on both sides, with some pollinators less willing to cooperate and act inside them (Howard et al. 2021). While we may never fully solve these issues and ability to generalise our results, we can be aware of those limitations when designing and conducting experiments, analysing results, drawing conclusions, and applying the knowledge to real-world scenarios or suggesting solutions to environmental or agricultural problems. This study provides a cautionary reminder that controlled behavioural choice experiments may not necessarily represent the behaviour and interactions observed in the real environment.

While the results of the current study are mixed, depending on field or laboratory conditions, the data suggests a need for further examinations of plant-pollinator relationships as a result of non-native species and more work into how honeybees may impact native pollinators as these factors can potentially result in significant environmental changes. It is an area of growing global interest and requires more research in Australia where there are many important endemic species and a history of largescale negative consequences from invasive species introductions.

## Data Availability

All data is available in the supplementary material.
